# Supplementary material for: Neutropenic sepsis and septic shock in ICU patients: A single-center experience over the last decade
Source: PLoS One. 2025 Oct 15;20(10):e0334511. doi: 10.1371/journal.pone.0334511 (PMC12527136; doi:10.1371/journal.pone.0334511)
Supplement: S1 File — (DOC) [file pone.0334511.s002.doc]

## S1 File: Definition of early source control

Source control refers to any procedure aimed at eradicating the source of sepsis. It includes removal of a potentially infected device (e.g. central lines), or definitive control of a source of ongoing microbial: surgical intervention for the removal of intra-abdominal abscesses, treatment of gastrointestinal perforation, ischaemic bowel or volvulus, cholangitis, cholecystitis, pyelonephritis associated with obstruction or abscess, removal of necrotizing soft tissue infection, other deep space infection (e.g., empyema or septic arthritis), and implanted device infections
